# Thoughts on Medical Reform

**Published:** 1834-01-01

**Authors:** 


*Thoughts on Medical Reform.* By A RETIRED PRACTITIONER. London, 1833. 8vo. pp. 33.

THIS is a temperate, sensible, well-timed pamphlet. Two points are discussed in it. The first is the necessity and justice of unbounded toleration in medicine, that is, of allowing every one to practise physic who can procure patients; and the second is, the expediency of establishing some new grades in the profession. To attempt to put down the unlicensed practitioner by the law, is to convert a quack into a martyr, and is a scheme worthy only of those who would endeavour to make the world happy by acts of parliament, and are quite sure that they could force the millennium by an unlimited supply of policemen and gallows. "Some members of the profession," says our author, "appear to consider the sick as their exclusive property, and to regard irregular practitioners as so many poachers or interlopers on their manor." To be sure they do; and they would establish a whole army of Johnsons and Byerses, by way of gamekeepers, to take care that the sick were made game of by none but a certificated person. But we are certain that the good sense as well as the dogged spirit of liberty existing in the English nation, will prevent these monstrous phantasms from being realized.

Our author is of opinion that there should be three grades of medical practitioners: 1st, Physicians and surgeons; 2dly, general practitioners; and 3dly, druggists. As the Scotch universities have reduced the value of an M.D. degree nearly to 0, he proposes that the members of the first class should be called Fellows of the Royal College of Medicine. He does not seem to see that no one of the first two classes ought to practise pharmacy; and he makes no mention of the host of nominal surgeons with which the London College has overspread the land, to the infinite detriment of the public, who continually mistake the man who has passed the college for a real surgeon, or, in other words, confound the legal and the medical surgeon.

